# Intestinal restriction of *Salmonella* Typhimurium requires caspase-1 and caspase-11 epithelial intrinsic inflammasomes

**DOI:** 10.1371/journal.ppat.1008498

**Published:** 2020-04-13

**Authors:** Shauna M. Crowley, Xiao Han, Joannie M. Allaire, Martin Stahl, Isabella Rauch, Leigh A. Knodler, Bruce A. Vallance

**Affiliations:** 1 Department of Pediatrics, BC Children’s Hospital, University of British Columbia, Vancouver, British Columbia, Canada; 2 Department of Medical Microbiology & Immunology, School of Medicine, Oregon Health & Science University, Portland, Oregon, United States of America; 3 Paul G. Allen School for Global Animal Health, College of Veterinary Medicine, Washington State University, Pullman, Washington, United States of America; University of Pennsylvania, UNITED STATES

## Abstract

We investigated the role of the inflammasome effector caspases-1 and -11 during *Salmonella enterica* serovar Typhimurium infection of murine intestinal epithelial cells (IECs). *Salmonella* burdens were significantly greater in the intestines of caspase-1/11 deficient (*Casp1/11*^−/−^), *Casp1*^−/−^ and *Casp11*^−/−^ mice, as compared to wildtype mice. To determine if this reflected IEC-intrinsic inflammasomes, enteroid monolayers were derived and infected with *Salmonella*. *Casp11*^−/−^ and wildtype monolayers responded similarly, whereas *Casp1*^−/−^ and *Casp1/11*^−/−^ monolayers carried significantly increased intracellular burdens, concomitant with marked decreases in IEC shedding and death. Pretreatment with IFN-γ to mimic inflammation increased caspase-11 levels and IEC death, and reduced *Salmonella* burdens in *Casp1*^−/−^ monolayers, while high intracellular burdens and limited cell shedding persisted in *Casp1/11*^−/−^ monolayers. Thus caspase-1 regulates inflammasome responses in IECs at baseline, while proinflammatory activation of IECs reveals a compensatory role for caspase-11. These results demonstrate the importance of IEC-intrinsic canonical and non-canonical inflammasomes in host defense against *Salmonella*.

## Author summary

Intestinal epithelial cells (IECs) are located at the interface between the gut lumen and the mucosal immune system and form the first layer of defense against the invasive enteric pathogen *Salmonella enterica* serovar Typhimurium. To prevent *Salmonella*, and other pathogens from establishing a foothold in the gut, the host mobilizes the inflammasome to selectively eject infected/compromised IECs from the epithelial layer into the intestinal lumen. This involves the activation of the inflammatory caspases; caspase-1 and -11. The individual contributions of each caspase to intestinal host defense, as well as the importance of IEC-intrinsic inflammasomes have not been previously defined, due to the lack of *Casp1*^−/−^ mice as well as appropriate IEC-intrinsic defense models. Here, we determined that both caspases contribute to controlling *Salmonella* pathogen burdens and IEC shedding in the mouse intestine. Caspase-1 appears to play a larger role at baseline since caspase-11 expression must be first induced through proinflammatory signalling. Our data also highlights that IEC-intrinsic caspase activation is sufficient for infection-induced cell shedding and that the intestinal epithelium is a key site for inflammasome-mediated immune defense.

## Introduction

Within the mammalian gastrointestinal (GI) tract, intestinal epithelial cells (IECs) provide the primary interface between the microbial-rich gut lumen and the underlying mucosal immune system. Here they play a central role in the coordination of mucosal homeostasis, tempering pro-inflammatory responses while remaining rapidly reactive to noxious stimuli such as enteric pathogens. One recently described mechanism by which IECs engage in immune defense is through the activation of cell-intrinsic inflammasomes that require inflammatory caspases, namely caspase-1 and caspase-11 in mice, or caspase-1 and caspase-4 in humans [1, 2].

During the initial stages of an enteric infection, *Salmonella enterica* serovar Typhimurium (*S*. Typhimurium) migrates from the gut lumen towards the intestinal epithelium, subsequently invading IECs. The invasion and intracellular proliferation of this pathogen triggers the activation of IEC-intrinsic inflammasomes, resulting in the expulsion of infected IECs into the intestinal lumen. The more rapidly these cells are shed, the less time is available for intracellular *Salmonella* to proliferate and invade surrounding IECs or translocate into the underlying lamina propria. In 2014, Sellin and colleagues showed this process requires the Nod-like receptors (NLRs) Naip1-6 and Nlrc4 [2], which form an inflammasome platform that activates caspase-1. During the early stages of a *S*. Typhimurium infection (12 h post-infection (p.i.)), the IECs lining the ceca of *Naip1-6*^−/−^ and *Nlrc4*^−/−^ mice were found to be heavily infected, containing densely packed microcolonies of intracellular *Salmonella* (up to 20 bacteria per cell), which were only rarely observed in the IECs of wildtype mice [2]. Through bone marrow transplantation studies, as well as the use of *Naip1-6*^*ΔIEC*−/−^ mice, the authors demonstrated this microcolony phenotype was caused by the loss of Naip-Nlrc4 inflammasome activation in IECs. Notably, the protective role for Naip1-6 was extremely acute as these mice were comparable to wildtype mice in *Salmonella* colonization of the cecum or histopathology at later time points (36 h p.i.)

In the study by Sellin *et al*., *Salmonella* loads in the mucosa of *Casp1/11*^*−/−*^ mice were between that of *Naip1-6*^−/−^ or *Nlrc4*^−/−^ mice and wildtype mice, whereas *Casp11*^*−/−*^ mice phenocopied wildtype mice at 18 h p.i. [2]. In an independent study, we demonstrated that a non-canonical inflammasome involving caspase-11 is activated at later time points during enteric *S*. Typhimurium infection in mice [1]. Specifically, *Casp11*^−/−^ mice carried higher *Salmonella* loads in the cecum and cecal lumen at 7 days p.i. and displayed an intracellular IEC microcolony phenotype similar to that described by Sellin *et al*. at 24 h p.i. [1]. Importantly, this phenotype was also observed in the gallbladder of *Casp11*^−/−^ mice, indicating that caspase-11 dependent control of epithelial cell shedding is not restricted to the intestine.

Thus the reports by Sellin *et al*. and Knodler *et al*. both detailed an important connection between epithelial-intrinsic inflammasome activation, cell shedding and intracellular bacterial burdens [1, 2]. However, the individual contributions and potential functional overlap of caspase-1 and caspase-11 to host protection against *Salmonella* in the gut has yet to be determined, primarily because mice deficient only in caspase-1 were not available. Recently this has changed, as *Casp1*^−/−^ mice have been generated by a handful of groups [3–5]. To define the exact involvement of caspase-1 and caspase-11 in antimicrobial defenses within the gut, we directly compared *S*. Typhimurium colonization in *Casp1*^−/−^, *Casp11*^−/−^ and *Casp1/11*^−/−^ mice as well as in enteroids. Our results demonstrate that caspase-1 primarily regulates inflammasome responses in IECs at baseline whereas caspase-11 plays a compensatory role upon extrinsic stimulation of inflammatory signaling pathways in IECs. Therefore, canonical and non-canonical IEC-intrinsic inflammasomes cooperate to provide an important innate immune defense against pathogen infections.

## Results

### Inflammasome-deficient mice carry higher intestinal tissue and luminal *Salmonella* burdens

**Fig 1:**
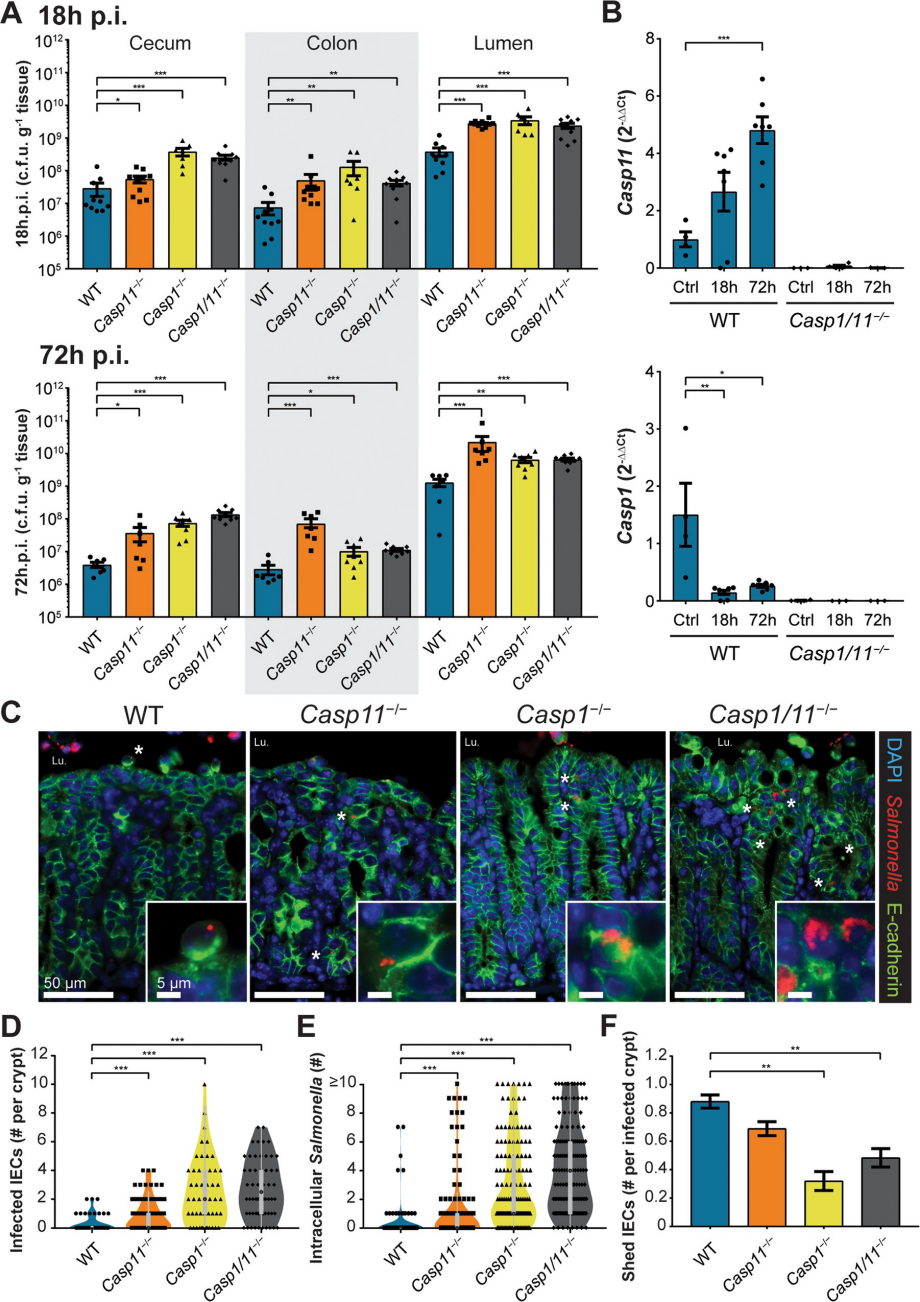
Inflammatory caspases are required for the epithelial restriction of a *Salmonella* infection *in vivo*. Streptomycin-pretreated C57BL/6 (WT), *Casp11*^−/−^, *Casp1*^−/−^ and *Casp1*^−/−^/*Casp11*^−/−^ (*Casp1*/*11*^−/−^) mice were orally infected with *S*. Typhimurium (3 × 10^6^ c.f.u.), with intestinal tissue and luminal contents plated at 18 h post infection (p.i.) and 72 h p.i. **(A)**. *Casp11* and *Casp1* gene expression enumerated relative to *Rplp0* reference from cecal RNA of streptomycin pretreated controls, 18 h p.i. and 72 h p.i., WT and *Casp1*/*11*^−/−^ mice. **(B)**. Representative fluorescence images of infected cecal tissues at 18 h p.i. *Salmonella* O-antigen (red), E-cadherin (green), and DNA (blue) **(C)**. Original magnification ×200, Inset ×630; scale bars 50 μm, inset scale bars 5 μm. Asterisk denotes presence of intracellular *Salmonella* (L.u. denotes cecal lumen). The number of *Salmonella*-infected IECs per a crypt **(D)**, the number of intracellular *Salmonella* in each infected IEC **(E)** and the proportion of apically shedding IECs adjacent to infected crypts **(F)**. Statistical significance for 1A and 1D-F calculated using Mann-Whitney U-test with student *t*-test applied to 1B (*p<0.05; **p<0.01; ***p<0.001). Each symbol represents one mouse. Mean and SEM are indicated. Results are from at least two independent experiments. Blinded 18 h p.i. cecal tissue analyzed in 1D-F were from n = 5 mice (with 50 representative crypts scored) from at least two independent experiments.

To define the exact contributions of caspase-1 and caspase-11 to enteric host defense, we infected C57BL/6 (wildtype; WT), *Casp1*^*−/−*^, *Casp11*^*−/−*^ and double-deficient *Casp1/11*^*−/−*^ mice with *S*. Typhimurium via the orogastric route. The *Casp1*^*−/−*^ and *Casp1/11*^*−/−*^ mice proved highly susceptible to infection, carrying heavy cecal, colonic and luminal pathogen burdens at 18 h p.i. (Fig 1A). Although their cecal tissue burdens were not as high as those carried by the *Casp1*^*−/−*^ and *Casp1/11*^*−/−*^ mice, the *Casp11*^*−/−*^ mice also displayed significantly higher intestinal and luminal burdens than WT mice at 18 h p.i. (*, P < 0.05, Fig 1A) and their intestinal burdens remained high at 72 h p.i. (Fig 1A). Interestingly, WT cecal burdens displayed a marked seven-fold decrease between 18 h and 72 h p.i. whereas only a minor decrease was observed in the *Casp1*^*−/−*^ and *Casp1/11*^*−/−*^ mice, while *Casp11*^*−/−*^ intestinal burdens remained comparable to those at 18 h p.i. This suggests the inflammatory caspase-deficient mice were unable to clear the infection from their tissues as efficiently as WT mice, a finding corroborated by their higher fecal shedding burdens (S1 Fig). Expression profiles of *Casp1* and *Casp11* in the cecal tissues of WT mice revealed that *Casp11* transcripts increased over the course of infection, while *Casp1* levels decreased (Fig 1B), which is consistent with other reports [2, 6, 7].

### Inflammasome-deficient mice display increased numbers of infected IECs and higher intracellular *Salmonella* burdens

To investigate if the increased intestinal burdens recovered from the caspase-deficient mice indicated potential differences in tissue localization, we used immunofluorescence staining of infected cecal tissues (18 h p.i.) to identify *S*. Typhimurium via its O-antigen and epithelial cells via epithelial cadherin (E-cadherin). In all mouse backgrounds, the majority of the *Salmonella* were confined to the cecal lumen, however a small intraepithelial (and intracellular) subset was also observed (Fig 1C). Focusing on this intracellular subset, we noted that the cecal crypts of WT mice remained relatively sterile, with only an occasional infected cell identified per crypt (fewer than 10% of crypts carried infected IECs at 18 h p.i.) (Fig 1D). Of the infected IECs, they contained only 1–2 *Salmonella* per cell on average (Fig 1E). Moreover, these infected WT IECs were largely confined to the tips of crypts or in the process of being actively shed from the epithelial surface (Fig 1C), similar to that described previously [1, 2]. In contrast, all the inflammatory caspase-deficient mice showed increased numbers of *Salmonella*-infected IECs (Fig 1C; 22%, 38% and 42% of the cecal crypts of *Casp11*^*−/−*^, *Casp1*^*−/−*^ and *Casp1/11*^*−/−*^ mice, respectively, showed infected IECs). *Casp1*^*−/−*^ and *Casp1/11*^*−/−*^ mice carried both the highest number of infected IECs/crypt (Fig 1D) (mean of 2–3 infected IECs/crypt), but also the highest number of *Salmonella* per IEC, with numerous IECs containing microcolonies comprising ≥10 bacteria (Fig 1E).

### Wildtype mice display high levels of IEC shedding largely localized to infected crypts

To address the connection between IEC shedding and *S*. Typhimurium invasion *in vivo*, 18 h p.i. cecal tissues were scored for epithelial damage. Whereas the ceca of WT mice demonstrated widespread signs of crypt and IEC deterioration, the cecal epithelium of *Casp1*^*−/−*^, *Casp11*^*−/−*^ and *Casp1/11*^*−/−*^ mice was largely intact, as were their cecal crypts (S2 Fig). While small numbers of shed IECs were found in the ceca of all the infected inflammatory caspase-deficient mice, the degree of IEC shedding was exaggerated in infected WT mice (Fig 1F; S2 Fig). For example, WT mice displayed severe erosion of their epithelial surface with increased IEC shedding at most crypt apical tips (S2 Fig). In contrast, the cecal epithelium of *Casp1*^*−/−*^ and *Casp1/11*^*−/−*^ mice demonstrated only minor desquamation whereas *Casp11*^*−/−*^ mice presented an intermediate phenotype, where the majority of crypts displayed only minor damage but with a modest increase in IEC shedding (S2 Fig). Of note, in WT mice there was a strong correlation between the presence of an infected crypt and local IEC shedding, while this relationship was largely lost in the ceca of the caspase-deficient mice (Fig 1F).

### *Casp1*^−/−^ and *Casp1/11*^−/−^ enteroid-derived monolayers exhibit increased numbers of infected IECs and higher intracellular *Salmonella* burdens

**Fig 2:**
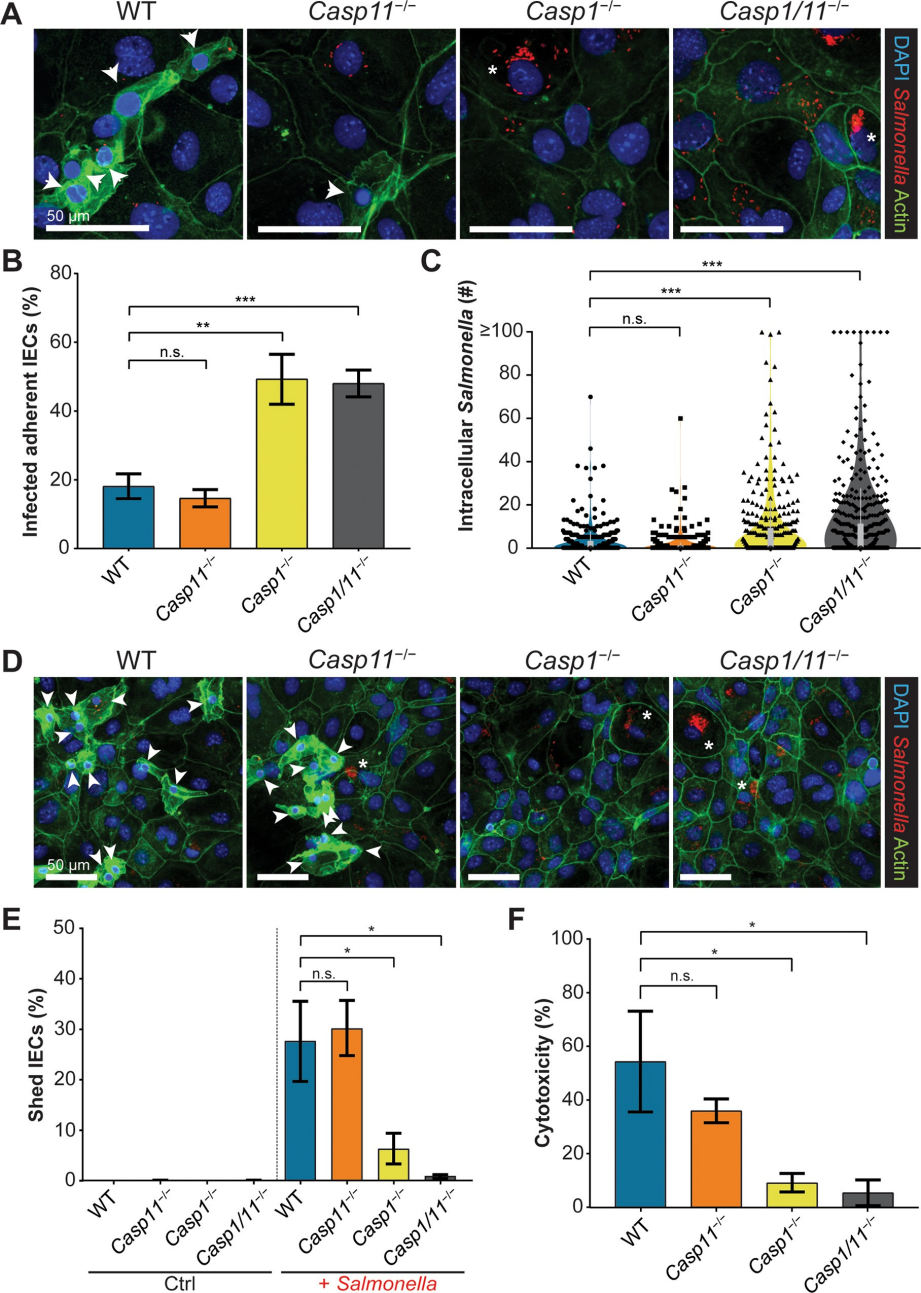
Epithelial intrinsic inflammasomes restrict the intracellular proliferation of *Salmonella* predominantly through caspase-1 induced cell shedding and death. Cecal enteroid monolayers derived from WT, *Casp11*^−/−^, *Casp1*^−/−^ and *Casp1*/*11*^−/−^ mice were infected with mCherry-*S*. Typhimurium (MOI of 50) and bacterial colonization was assessed at 10 h p.i. by fluorescence microscopy. Representative fluorescence images depicting *Salmonella* (red), actin (green) and DNA (blue) **(A)**. Original magnification ×400; scale bars 50μm. Arrows denote actively shedding or shed IECs and asterisks denote large foci of intracellular *Salmonella*. The severity of infection was also determined by the percentage of adherent infected IECs **(B)** and the number of intracellular *Salmonella* in each infected IEC **(C)**. Results are from at least 400 IECs from two independent experiments. Representative fluorescence images of *Salmonella*-induced cell shedding; *Salmonella* (red), actin (green) and DNA (blue) **(D)**. Original magnification ×200; scale bars 50μm. Percentage of shed/shedding IECs from the monolayer (from at least four blinded fields of view from two independent experiments) **(E)**. IEC cytotoxicity as measured by lactate dehydrogenase activity released into the growth media at 10 h p.i. **(F)**. Mean and SEM indicated from duplicate wells in two independent experiments. Statistical significance for 2B-C and 2E calculated using Mann-Whitney U-test and student *t*-test applied to 2F (n.s. p>0.05; *p<0.05; **p<0.01; ***p<0.001).

To define whether the ability of inflammatory caspases to restrict *S*. Typhimurium infection in murine ceca reflected an IEC intrinsic role, or alternatively, confounding factors such as the intestinal microbiota or infiltrating immune cells that may alter IEC function, we generated cecal enteroids from uninfected mice. After generating 2D monolayers from these enteroids, they were infected with mCherry-*S*. Typhimurium (wildtype bacteria constitutively expressing the fluorescent protein, mCherry) and intracellular bacteria were enumerated using a gentamicin protection assay in combination with fluorescence microscopy. *Casp1*^*−/−*^ and *Casp1/11*^*−/−*^ monolayers proved highly susceptible to *Salmonella* infection and the majority of infected IECs remained intact within the monolayer (Fig 2A and 2B). Moreover, many of these infected cells contained large microcolonies of intracellular *Salmonella*, with some IECs containing over 100 bacteria at 10 h p.i. (Fig 2C). In contrast, WT and *Casp11*^*−/−*^ monolayers (Fig 2A–2C) showed stronger responses to infection, with significantly fewer infected adherent IECs (Fig 2B) as well as relatively low numbers of intracellular *Salmonella* (Fig 2C). This result is consistent with a previous report that caspase-11 is not required for *S*. Typhimurium restriction within IECs *in vivo* [2]. With the exception of the *Casp11*^*−/−*^ monolayers, intracellular burdens in enteroids largely mirrored the *in vivo* findings that inflammatory caspase-deficient mice carried higher intracellular *Salmonella* levels than WT mice (Fig 2C). Bacterial colony forming unit (CFU) counts from infected monolayers at 10 h p.i. mirrored trends revealed in the fluorescence microscopy analysis described above (S3 Fig). Moreover, no significance difference in intracellular CFU was detected when comparing infection with wildtype *S*. Typhimurium and mCherry-*S*. Typhimurium (S3 Fig).

### Wildtype and *Casp11*^−/−^ enteroid-derived monolayers display increased cell shedding and death

We have previously observed the shedding of *Salmonella*-infected IECs *in vivo* and *in vitro* [1, 8]. Shedding IECs were also evident in our murine enteroid infection model, in agreement with a recent study of *S*. Typhimurium infection of human enteroids [9]. Compared to adherent IECs, shedding IECs presented a markedly different cell morphology; characterized by small, condensed DNA (leading to a comparatively stronger DAPI/nuclei signal), a ‘ruffled’ cytoskeletal actin signal and a slightly higher z-axis location in the monolayer, whereas adherent IECs displayed larger nuclei and clear cell-to-cell junction ‘lattice-like’ actin staining (Fig 2A and 2D) [8]. To quantify cell shedding, the size and intensity of DAPI signals were evaluated. IEC shedding was found to be significantly enhanced upon bacterial infection in WT and *Casp11*^*−/−*^ monolayers as compared to modest shedding in *Casp1*^*−/−*^ and *Casp1/11*^*−/−*^ cells (Fig 2D and 2E). The proportion of infected, shed IECs compared to infected, adherent IECs was also comparatively higher in WT and *Casp11*^*−/−*^ monolayers (S4 Fig). Similarly, infected WT and *Casp11*^*−/−*^ monolayers exhibited higher levels of cytotoxicity compared to *Casp1*^*−/−*^ and *Casp1/11*^*−/−*^ monolayers, as measured by the release of the cytosolic enzyme, lactate dehydrogenase, into the growth media (Fig 2F). These results support the concept that inflammatory caspases promote the expulsion of infected, dying IECs into the gut lumen [1, 2, 8].

### Inflammatory caspase activity is present in *Salmonella*-infected shedding cells and not detectable in *Casp1/11*^−/−^ monolayers

**Fig 3:**
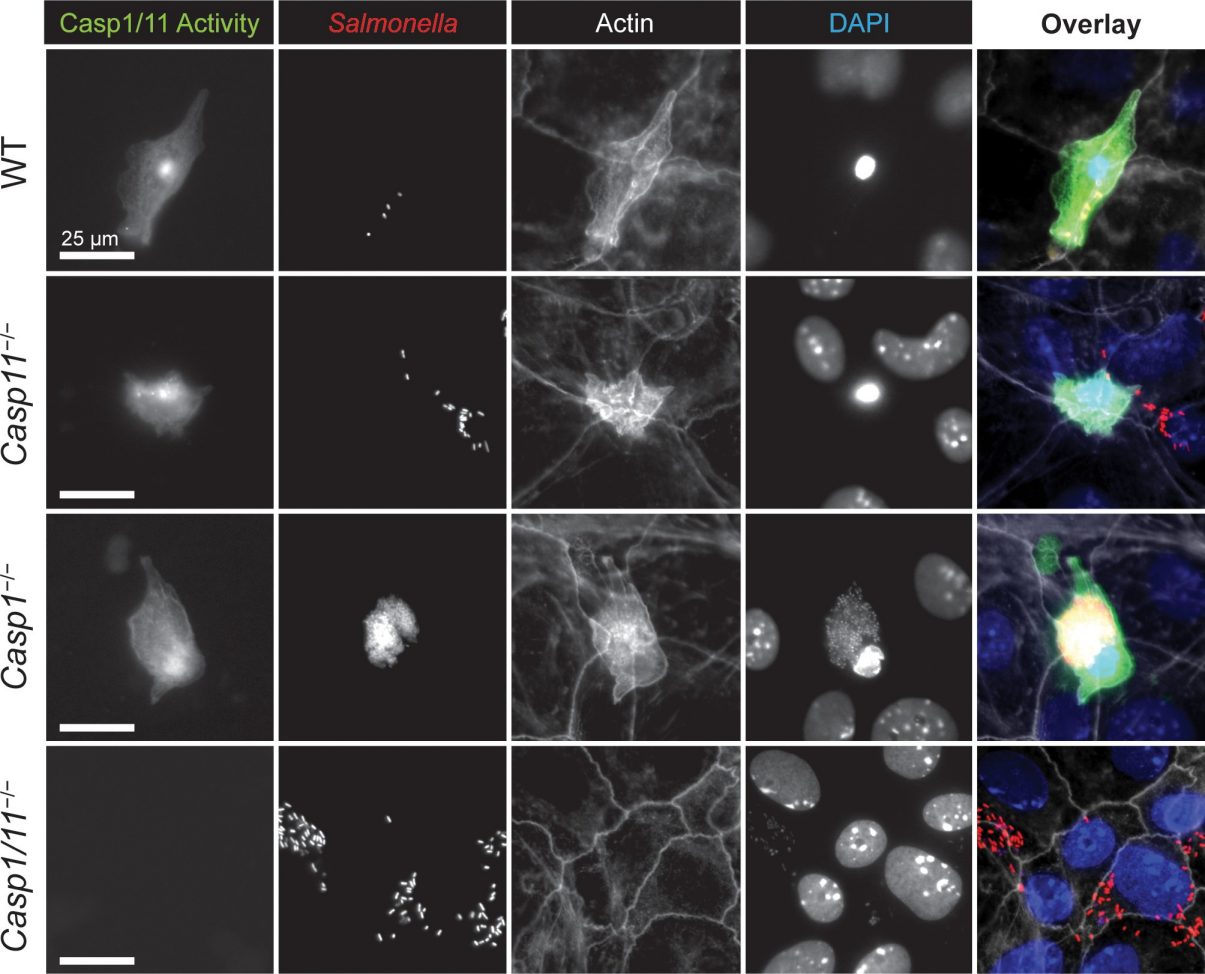
Shedding IECs have active inflammatory caspases. Cecal enteroid monolayers generated from WT, *Casp11*^−/−^, *Casp1*^−/−^ and *Casp1*/*11*^−/−^ mice were infected with mCherry-*S*. Typhimurium (MOI of 50) and assessed for inflammatory caspase activity at 10 h p.i. Representative fluorescence images depicting active caspase-1/11 (660-YVAD-FMK probe; green), *Salmonella* (red), actin (white) and DNA (blue). Original magnification ×400; scale bars 25 μm.

We previously showed that *Salmonella*-infected human IECs undergoing extrusion have active inflammatory caspases [8]. To detect inflammatory caspase activity in infected murine enteroids we employed a cell permeable fluorescent caspase activity dye (660-YVAD-FMK FLICA), which covalently couples to active caspase-1 and/or caspase-11. By fluorescence microscopy, WT monolayers exhibited a strong fluorescence signal specifically in infected cells undergoing shedding, indicating active caspase-1/11 (Fig 3). This phenotype was also seen in *Casp1*^*−/−*^ and *Casp11*^*−/−*^ monolayers, despite overall lower numbers of shedding IECs in the *Casp1*^*−/−*^ monolayers. In contrast, active caspase-1/11 was not detected in the *Casp1/11*^*−/−*^ monolayers (S5 Fig), reinforcing the specificity of the FLICA probe for caspase-1 and -11. The FLICA signal in WT, *Casp1*^*−/−*^ and *Casp11*^*−/−*^ monolayers appeared diffuse throughout the cell cytoplasm, while small high-intensity puncta were also observed in WT and *Casp11*^*−/−*^ monolayers (Fig 3), appearing similar to the FLICA-positive signals described for *Nlrc4-*canonical inflammasome formation in macrophages [10]. These results suggest that caspase-1 activity is dominant in IECs, but a caspase-11 inflammasome is also present, albeit more evident in cells lacking caspase-1 function.

### IFN-γ priming functionally differentiates enteroid monolayers derived from *Casp1*^−/−^ and *Casp1/11*^−/−^ mice in terms of *Salmonella*-induced cell shedding

**Fig 4:**
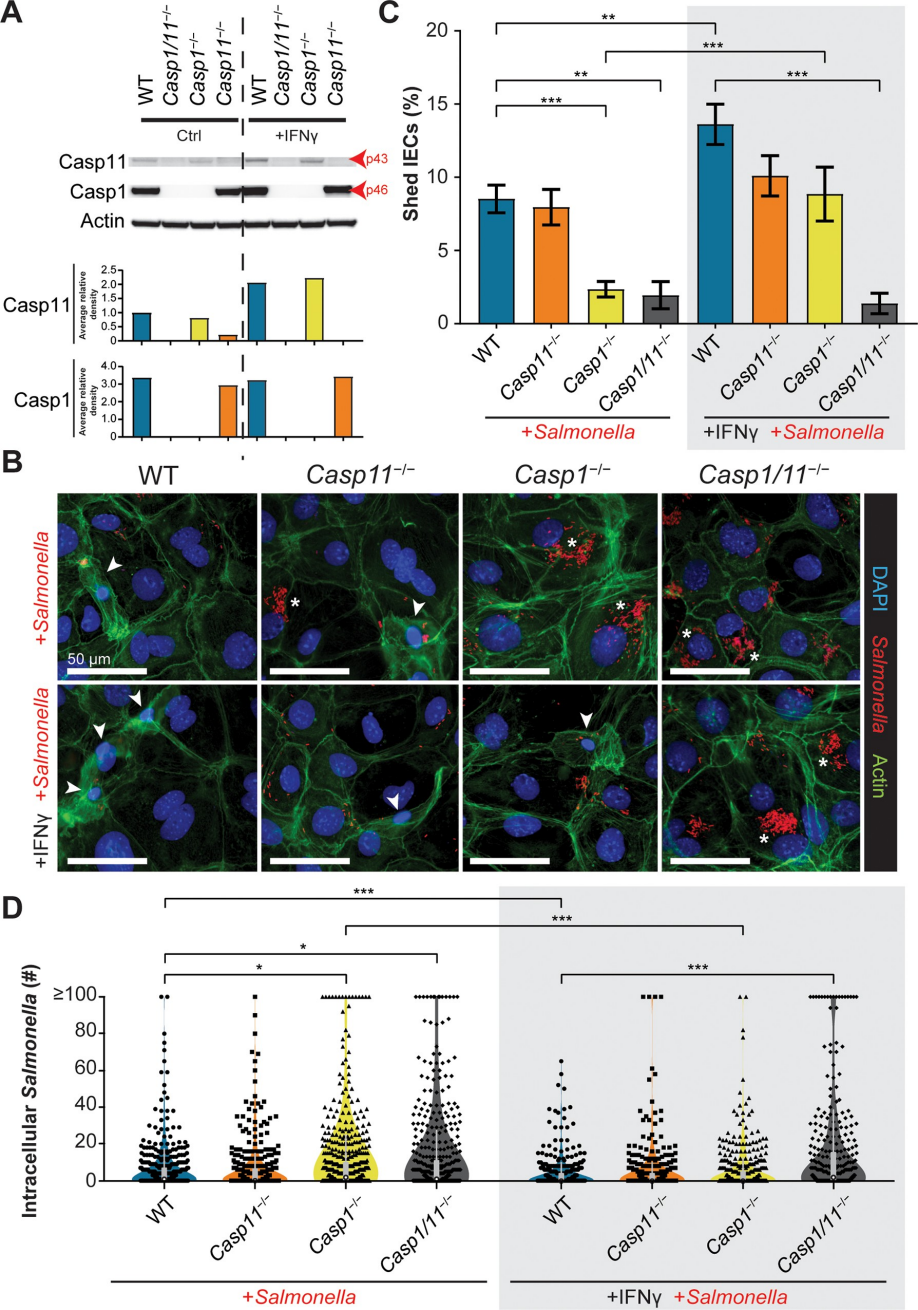
IFN-γ pretreated enteroid monolayers display enhanced levels of IEC shedding and a dependence on caspase-1 and caspase-11 for restriction of *Salmonella* infection. Enteroids generated from WT, *Casp11*^−/−^, *Casp1*^−/−^ and *Casp1*/*11*^−/−^ mice were treated for 16 h with vehicle control or IFN-γ (10 ng/mL), then cell lysates probed for pro-caspase-1 (p46), pro-caspase-11 (p43) or actin by Western blotting. Densities relative to actin are shown **(A).** Data from a representative experiment is presented. Three independent experiments gave similar results. Cecal enteroid monolayers from WT, *Casp11*^−/−^, *Casp1*^−/−^ and *Casp1*/*11*^−/−^ mice were either pretreated with IFN-γ (10 ng/mL) or vehicle control 16 h prior to infection with mCherry-*S*. Typhimurium (MOI of 50). Representative fluorescence images depicting *Salmonella* (red), actin (green) and DNA (blue) of cecal enteroid monolayers at 10 h p.i. either with IFN-γ or vehicle control pretreatment **(B)**. Original magnification ×400; scale bars 50μm. Arrows denote actively shedding or shed IECs and asterisks denote large foci of intracellular *Salmonella*. Percentage of shed/shedding IECs (from five blinded fields of view from two independent experiments) **(C)**. The number of intracellular *Salmonella* in each infected IEC either primed with IFN-γ or vehicle control **(D)**. Results are from 400 IECs from two independent experiments. Statistical significance for 4C and 4D calculated using Mann-Whitney U-test (*p<0.05; **p<0.01; ***p<0.001).

We hypothesized that the limited role for caspase-11 in enteroid-derived monolayers could be due to a lack of extrinsic factors present during *in vivo* infection of mice, such as inflammatory cytokines and chemokines that directly modulate IEC expression of innate defense proteins [11]. Interferon (IFN)-γ is a potent cytokine released by immune cells which induces hundreds of genes promoting host defense [7, 12]. It helps drive mucosal inflammation during the late stages of *Salmonella*-induced colitis and has been described as an early stage effector cytokine with high systemically circulating levels during the first day of oral infection [13, 14]. When we analyzed cecal tissues collected from streptomycin-pretreated uninfected and *S*. Typhimurium-infected mice at 18 h and 72 h p.i. we noted that IFN-γ protein levels were significantly elevated in all infected genotypes at 18 h p.i., as compared to uninfected control tissues (S6 Fig). Interestingly, IFN-γ levels remained high in the infected ceca of WT mice at 72 h p.i., but significantly lower levels were produced by the inflammatory caspase-deficient mice, particularly the *Casp1*^*−/−*^ and *Casp1/11*^*−/−*^ mice. To test whether IFN-γ could alter inflammatory caspase production in IECs, pro-caspase-1 and pro-caspase-11 levels in naïve and IFN-γ treated WT cecal enteroids were compared by immunoblotting. High levels of caspase-1 were present irrespective of IFN-γ treatment, whereas caspase-11 levels increased upon IFN-γ treatment (Fig 4A). This suggests that the activity of the non-canonical inflammasome might be potentiated in murine IECs as part of the host inflammatory response to infection.

Next, we assessed the effect of IFN-γ-priming on the enteroid monolayer response to infection. IFN-γ pretreatment alone had no overt effect on cell shedding under basal conditions for all genotypes (S7 Fig). However, IFN-γ pretreatment followed by *S*. Typhimurium infection significantly increased cell shedding in WT and *Casp1*^*−/−*^ enteroid monolayers (Fig 4B and 4C). In contrast, shedding of infected *Casp11*^*−/−*^ and *Casp1/11*^*−/−*^ monolayers was not affected by IFN-γ priming, with *Casp11*^*−/−*^ monolayers maintaining high levels of cell shedding that were not significantly different from its untreated, infected control, while *Casp1/11*^*−/−*^ monolayers exhibited little IEC shedding even after pretreatment with IFN-γ (< 2% of total IECs) (Fig 4B and 4C).

We next assessed if increased IEC expression of caspase-11 upon IFN-γ pretreatment impacted *S*. Typhimurium infection. Mirroring the cell shedding phenotype, IFN-γ priming decreased the mean number of intracellular *Salmonella* per cell in WT and *Casp1*^*−/−*^ enteroid monolayers, compared to no IFN-γ treatment (Fig 4B and 4D). Strikingly, IFN-γ pretreatment of *Casp1*^*−/−*^ monolayers nearly eliminated intracellular *Salmonella* microcolony formation (≥100 bacteria/cell) to less than 1% of all infected IECs (Fig 4D). By contrast, IFN-γ pretreatment did not alter the mean number of intracellular *Salmonella* per cell for *Casp11*^*−/−*^ and *Casp1/11*^*−/−*^ monolayers (Fig 4B and 4D). Overall, these findings indicate that extrinsic stimuli, such as IFN-γ, promote caspase-11, but not caspase-1 function in IECs.

## Discussion

Through the use of *in vivo* mouse infections as well as enteroid-derived monolayers, we have clarified the contributions of caspase-1 and caspase-11 to the IEC-intrinsic inflammasome and its ability to restrict enteric *Salmonella* infections. While previous work compared responses in *Casp11*^*−/−*^ and *Casp1/11*^*−/−*^ mice [1, 2], here using *Casp1*^*−/−*^ and *Casp11*^*−/−*^ mice and enteroids, we unequivocally demonstrate that IECs utilize both inflammatory caspases to launch an intrinsic multilayered innate defense. In this study, we found that both caspase-1 and caspase-11 are required to effectively control an enteric *S*. Typhimurium infection. Caspase-1 dominates the antimicrobial response to *Salmonella* at early time points, while caspase-11 mediated defense plays a larger role later in the course of infection. This timeline agrees with our previous observations that *Casp11*^*−/−*^ mice carried significantly higher cecal burdens than WT mice at 7d p.i. with the *S*. Typhimurium Δ*aroA* strain [1].

Enteroids offer an attractive alternative to traditional cell culture, providing a physiologically relevant model system derived from the genotypic tissue of interest [15]. The use of enteroid-derived monolayers enables the study of bacterial-IEC interactions on various genetic backgrounds without the requirement for expression knockdowns prior to infection [16]. In murine cecal-derived enteroids, we showed that under baseline conditions IEC-intrinsic caspase-1 plays the major role in restricting intracellular *Salmonella* proliferation (Fig 2). In contrast, after pretreatment with IFN-γ to mimic the inflammatory environment that develops during an *in vivo* infection, high pathogen loads only developed in *Casp1/11*^*−/−*^ monolayers (Fig 4), indicating that both caspase-1 and caspase-11 exert potent antimicrobial responses within “inflamed” IECs.

Studies have previously shown that murine caspase-1 is highly expressed in naïve tissues whereas expression of caspase-11 requires pro-inflammatory induction, potentially in response to activation of the NF-κB, IRF3 or STAT pathways [2, 6, 7]. At the beginning of a *S*. Typhimurium infection *in vivo*, baseline tissue expression of *Casp11* is low but is upregulated over the course of infection, whereas *Casp1* transcript levels are high at baseline and decline slightly as the infection progresses [2]. This expression profile is corroborated by our finding that levels of pro-caspase-11, but not pro-caspase-1, increased in IECs in response to IFN-γ treatment. In a recent publication by Hausmann *et al*., it was reported that TNF-α treatment of murine small intestine-derived enteroids also increased *Casp11* expression [17]. Together with our findings, this suggests that although caspase-1 is sufficient to protect naïve IECs, as the infection proceeds, host inflammatory responses upregulate caspase-11 expression in IECs, leading to increased caspase-11 activity; the combined efforts of these two caspases form a multilayered innate defense that controls intracellular *Salmonella* burdens and protects the host from pathogen attack.

Notably, our *in vivo* mouse studies show that both caspase-1 and caspase-11 are necessary for optimal host defense against *S*. Typhimurium (Fig 1). While our enteroid studies also show a clear role for both caspases *in vitro*, expression of either caspase was sufficient to promote inflammasome-mediated control over *Salmonella* expansion in murine IECs, including the expulsion of infected IECs from monolayers. These differences likely reflect a role for the inflammatory milieu in fine-tuning IEC inflammasome function as well as the contribution of inflammatory caspase-mediated activity from other cell types. Caspase-11 expression by IECs and macrophages needs to be induced by proinflammatory signaling before it can provide protection against *S*. Typhimurium [2, 4, 6, 7]. Thus, the impaired IFN-γ expression displayed by infected inflammatory caspase-deficient mice may have been insufficient to induce caspase-11 expression to levels sufficient to protect *Casp1*^*−/−*^ mice.

A vital innate defense phenotype mediated by the IEC intrinsic inflammasomes is cell shedding accompanied by pyroptosis [8]. Correspondingly, we found that loss of inflammatory caspase signaling in murine enteroids led to decreased cell shedding and resulted in a heavily infected, but relatively intact, monolayers. Defective inflammasome signaling in these monolayers also led to large intracellular microcolonies of *Salmonella*, as compared to the relatively small intracellular burdens seen in WT monolayers. We have previously reported similar findings during *S*. Typhimurium infection of human IEC lines [1]. We propose that these increased bacterial burdens are due to the inability of *Casp1/11*^*−/−*^ monolayers to expel infected, pyroptotic IECs. However, in macrophages, there is also an inflammasome-mediated restriction of *Salmonella* intracellular growth that occurs prior to cell lysis, that is both caspase-1 and caspase-11 dependent, but independent of gasdermin D (Gsdmd) [18]. The identity(s) of the cytosolic antimicrobial agent(s) mediating this protective effect remains unknown, but based on our study design, we cannot rule out its contribution to overall inflammasome-dependent intracellular *Salmonella* restriction. Interestingly, a low level of cell shedding was observed at 10 h p.i. in *Casp1/11*^*−/−*^ monolayers suggesting there is a minor inflammatory caspase-independent IEC expulsion component in our enteroid infection model. Previous work found that treatment of *Casp1*^*−/−*^ ileal enteroids with FlaTox (a cytoplasmic delivery reagent of Naip5 ligand) induced IEC expulsion that was independent of plasma membrane disruption [3]. Through the use of murine knockout and inducible expression models, it was demonstrated that caspase-8, in the absence of caspase-1, can induce IEC cell expulsion in an Asc-dependent manner [3]. Thus, although the limited number of shed IECs we noted in the *S*. Typhimurium-infected *Casp1/11*^*−/−*^ monolayers did not exhibit caspase-1/11 activity, they may be extruded via this caspase-8 dependent mechanism.

We also observed a significant increase in the number of shedding IECs in WT and *Casp1*^*−/−*^ enteroid-derived monolayers upon IFN-γ pretreatment. We hypothesize this increase is due to IFN-γ increasing caspase-11 expression, since no significant changes in cell shedding were demonstrated by *Casp11*^*−/−*^ or *Casp1/11*^*−/−*^ monolayers upon IFN-γ pretreatment. In macrophages, pro-caspase-11 is present at very low levels under resting conditions and can be induced through type I interferon (IFN-α and IFN-β) and type II interferon (IFN-γ) treatment [4, 6, 19, 20]. Interestingly, complement signalling also amplifies caspase-11 activity in macrophages via upregulation of *Casp11* transcript [21]. While transcriptional induction is one aspect of caspase-11 activity, IFN-γ signalling also potentiates non-canonical (caspase-11) inflammasome activation by inducing the expression of guanylate binding proteins (GBPs). These can control macrophage antibacterial immune responses in both inflammasome-dependent and -independent manners and are known to be expressed in IECs [22, 23]. The GBPs potentiate caspase-11 based pyroptosis through the disruption of pathogen-containing vacuoles and since caspase-11 is an LPS sensor, the GBP-enhanced release of LPS into the cytosol augments non-canonical inflammasome activation [23–26].

Interestingly, the two inflammatory caspases are required for the rapid induction of IFN-γ in the gut during the early stages of *S*. Typhimurium infection (12 h p.i.) [27]. However, the impact of IFN-γ on pathogen burdens and mucosal inflammation is not evident until 48 h p.i. [13, 14, 28]. Acute sources of IFN-γ include innate lymphoid cells, NK cells, neutrophils and intestinal intraepithelial lymphocytes [13, 14, 28–31]. Interestingly, Songhet *et al*. observed that control over *S*. Typhimurium burdens within IECs was IFN-γ dependent, and although bone marrow-derived IFN-γR signaling controlled systemic spread, stromal IFN-γR expression was required for this control in IECs [28]. Based on our findings, it appears that caspase-11 plays a larger role during the later stages of an acute murine infection, after its expression has been induced by proinflammatory signals such as IFN-γ. To study the contribution of IEC-intrinsic caspase-11 to overall host defense and the interplay of IFN-γ signaling, further infection studies using conditional knockout mice, cell culture, enteroids or less virulent strains of *Salmonella* (e.g. Δ*aroA*) as well as the evaluation of other cytokines induced during *Salmonella* infection will be required [32].

In conclusion, our study defines the importance of the IEC-intrinsic inflammasomes in early host restriction of a *S*. Typhimurium infection. We hypothesize that caspase-1 drives the initial inflammasome mediated antimicrobial response through Nlrc4 mediated detection of Naip ligands; *Salmonella* pathogenicity island-1 (SPI-1) type III secretion system (T3SS) needle components (PrgJ and PrgI) and flagella (FliC) [2, 33–35]. Upon inflammatory caspase activation, pyroptosis and cell extrusion is initiated, expelling the compromised IEC into the intestinal lumen. By sacrificing these infected cells, the epithelium maintains its sterility, disables the ability of *Salmonella* to expand their infective niche, meanwhile secreting cytokines and chemokines to recruit professional immune cells to further bolster host defenses. These proinflammatory pathways in turn induce *Casp11* expression and newly primed IECs can now more effectively detect intracellular *Salmonella* through caspase-11 LPS recognition as well as identify those bacteria which have evaded Naip detection through downregulation of SPI-1 and/or *fliC* expression [36–38]. These actions highlight the complex and critical roles played by the intestinal epithelium in dealing with invasive bacterial pathogens, such as *Salmonella enterica*.

## Materials and methods

### Ethics statement

All mouse experiments, maintenance and care were performed according to protocols approved by the University of British Columbia's Animal Care Committee (Permit Number: A15-0211) and in direct accordance with the Canadian Council on Animal Care (CCAC) guidelines. Animals were weighed and monitored daily to ensure animal welfare.

### Mouse strains and infections

*Casp11*^*−/−*^ and *Casp1/11*^*−/−*^ (*Ice*^*−/−*^ or *Casp1*^*−/−*^ *Casp11*^*null/null*^) mice were obtained from Genentech [19]. *Casp1*^*−/−*^ mice have been described previously [3]. Female C57BL/6 (WT) and the various inflammatory caspase-deficient mice were used at 8–12 weeks old and bred under specific pathogen-free conditions at the BC Children’s Hospital Research Institute. For oral infections, mice were gavaged with streptomycin (100 mg/kg) 24 h before infection, then orally gavaged with an overnight LB culture of wildtype *S*. Typhimurium SL1344 (naturally streptomycin resistant, [39]) diluted in PBS (~3 × 10^6^ CFU) and euthanized at 18 h or 72 h p.i.

### Tissue collection and bacterial counts

Mice were anesthetized with isoflurane and euthanized via cervical dislocation. For *S*. Typhimurium enumeration, the cecum, colon and combined cecal and colonic luminal contents were collected and homogenized separately in 1 mL of sterile PBS. Samples were serially diluted and plated onto streptomycin-supplemented LB agar plates and incubated at 37°C overnight. Colonies were then enumerated and normalized to tissue weights. Tissue samples for histology and immunostaining were fixed in 10% neutral buffered formalin (Fischer Scientific) overnight then transferred to 70% ethanol. All fixed tissue was embedded in paraffin and cut into 5 μm sections.

### Immunofluorescent staining of infected tissues

Immunofluorescent staining proceeded as outlined previously [1]. In brief, paraffin embedded tissues were deparaffinized by heating to 60°C for 15 min, cleared with xylene, and rehydrated through an ethanol gradient to water. Antigen retrieval was performed in steam heated citrate buffer for 30 mins, before cooling to room temperature and washing with water. Tissues were treated in PBS, 0.1% Triton X-100 and 0.05% Tween 20 for 15 mins, then blocked with 5% donkey serum in PBS, 0.01% Triton X-100 and 0.05% Tween 20. Primary antibodies used were *Salmonella* O antisera Group B (Factors 1, 4, 12, 27) (1:1000, BD) and anti-E-cadherin (1:100; BD Biosciences). Tissues were then probed with Alexa Fluor 488-conjugated donkey anti-goat IgG (1:1000; Life Technologies) and Alexa Fluor 568-conjugated donkey anti-rabbit IgG (1:2000; Life Technologies). Tissues were mounted using ProLong Gold Antifade reagent (Life Technologies) containing DAPI for DNA staining. Sections were viewed on a Zeiss AxioImager microscope and images taken using an AxioCam HRm camera operating through AxioVision software.

### Intracellular *Salmonella quantification in vivo*

Cecal sections that had been immunostained for *Salmonella*, E-cadherin and DAPI, were blinded and manually studied at a magnification of ×400 to enumerate infected IECs per crypt, the number of intracellular *Salmonella* in IECs, and the presence of IEC(s) apically shedding from infected crypts (a score of 1 was given when shed IEC(s) were present, while 0 was awarded when no adjacent shedding IEC was present). For all enumerations, five separate cross sections from each mouse background were used, from two or more independent experiments and ten non-adjacent crypts for each cross section were selected. Epithelial integrity for the entire cross section were also evaluated as described by Barthel *et al*. with modification [40, 41] (0, no pathological changes detectable; 1, epithelial desquamation [a few cells shed, surface rippled]; 2, erosion of epithelial surface [epithelial surface rippled, damaged]; 3, epithelial surface severely disrupted/damaged, large amounts of cell shedding).

### Generation of cecal enteroids

Enteroids from murine ceca were isolated from each mouse background as previously described [15, 16, 42]. In brief, the ceca were excised, the tip and base removed, laterally opened to expose the apical surface, while luminal contents were removed and placed in Advanced DMEM/F12 (Gibco) supplemented with Pen Strep (100 U/ml, Gibco) and gentamicin (50 μg/ml, Gibco) on ice. The tissue was washed ten times in ice-cold Advanced DMEM/F12 (Gibco) with extensive vortexing, then transferred to Cell Recovery Solution (Corning) and incubated on ice for 30 mins. Under sterile conditions, forceps were used to gently liberate cecal crypts from the underlying tissue and the remaining tissue was discarded. The solution containing the cecal crypts was then centrifuged and washed twice with base media (Advanced DMEM/F12, Gibco) supplemented with Pen Strep (100 U/ml, Gibco), GlutaMAX (1X, Gibco) and HEPES (0.01 M, Gibco)) then diluted 1:1 in Matrigel (Corning). This was pipetted into several ‘domes’ on a 24-well plate and incubated at 37°C with 5% CO_2_. After the Matrigel solidified, growth media (base media supplemented with 1X condition media from L-WRN cells (CRL-3276, ATCC), N2 (Invitrogen), B27 (Invitrogen), N-acetylcystine (Sigma-Aldrich), nicotinamide (Sigma), mEGF (Invitrogen), A 83–01 (Tocris), SB 202190 (Sigma-Aldrich), and Y-27632 (Abmole)) was added to the well and incubated at 37°C with 5% CO_2_ [15]. L-WRN cells were cultured as previously described and condition media collected every 48h [15]. Media was changed every three days (growth media without Y-27632 supplementation) and the enteroids were passaged every five to seven days. For IFNγ-treated enteroids, growth media was supplemented with murine IFNγ (10 ng/mL; Peprotech) or corresponding volume of growth media for 16 h.

### Enteroid monolayer seeding and *Salmonella* infection of monolayers

Monolayers were generated as outlined previously [16, 43] with modifications. First, the growth media was removed, then four Matrigel ‘domes’ were pooled and disrupted through the addition of ice-cold Cell Recovery Solution and incubation on ice for 30 mins. Enteroids were then centrifuged and washed twice with base media, resuspended in Trypsin-EDTA (0.05%, Gibco) and incubated at 37°C with 5% CO_2_ for 10 mins. Enteroids were then mechanically disrupted into single cell suspensions with repeated pipetting through a p200 tip, and an equal volume of monolayer media (base media supplemented with N2 (Invitrogen), B27 (Invitrogen), and Y-27632 (Abmole) was added. Cells were centrifuged, then resuspended in monolayer media and added dropwise to Geltrex (Gibco) coated coverslips in 24-well plates. Monolayers were incubated at 37°C with 5% CO_2_ and media changed 24 h after seeding. Confluent monolayers were infected 72 h after seeding.

*S*. Typhimurium SL1344 WT *glmS*::*Ptrc-mCherryST* [1] was grown overnight in LB (5g/L NaCl) at 37°C then diluted 1:300 into 10 mL of LB (Miller; 10g/L NaCl) and grown for 4 h at 37°C with shaking [8]. The culture was then centrifuged, washed in PBS then diluted in infection media (monolayer media without Pen Strep). *Salmonella* was added to the monolayers at a MOI of 50:1 (bacteria:eukaryotic cell) and incubated at 37°C with 5% CO_2_ for 10 mins, then washed three times with PBS, and fresh infection media added for 20 mins. Monolayers were then treated with 50 μg/mL of gentamicin for 40 mins at 37°C with 5% CO_2_. Media was discarded then fresh infection media supplemented with 10 μg/mL of gentamicin added and monolayers incubated at 37°C with 5% CO_2_ for a total infection period of 10 h. After infection, two 50 μl aliquots of media from each condition were transferred to a black bottom 96-well plate for LDH activity quantification through the CytoTox-ONE Homogeneous Membrane Integrity Assay (Promega) performed according to manufacturer’s instructions. Monolayers were washed three times with PBS, fixed in 4% paraformaldehyde (PFA, Thermo Scientific) in the dark at RT for 30 mins, then used for immunostaining. For CFU counts, following the 10 h infection monolayers were lysed with 0.2% (w/v) sodium deoxycholate PBS solution for 10 mins with orbital shaking (400 rpm) at RT, lysates plated on LB and colonies enumerated the next day.

### Cell shedding, infection and intracellular *Salmonella* quantification of enteroid monolayers

PFA-fixed enteroids on coverslips were treated in PBS, 0.1% Triton X-100 and 0.05% Tween 20 for 15 mins, then blocked with 2% donkey serum in PBS, 0.01% Triton X-100 and 0.05% Tween 20 overnight. Coverslips were then stained with Alexa Fluor 488-phalloidin (1:2000; Life Technologies) for 30 mins, washed and mounted using ProLong Gold Antifade reagent (Life Technologies) containing 4′,6-diamidino-2-phenylindole (DAPI) for DNA staining. For determination of inflammatory caspase activity, the staining proceeded as outlined by Knodler *et al*. [8]. One hour prior to the end of infection, 660-YVAD-FMK (Immunochemistry Technologies) was diluted 1:30 into infection media and incubated at 37°C with 5% CO_2_ for 1 h, and further prepared according to manufacturer’s instructions, before incubation with Alexa Fluor 488-phalloidin and mounted onto glass slides using ProLong Gold Antifade reagent. Sections were viewed on a Zeiss AxioImager microscope and images taken using an AxioCam HRm camera operating through AxioVision software.

Fixed enteroid monolayers were blinded and images at a magnification of ×200 (shedding) or ×400 (infection/intracellular *Salmonella*) were obtained then evaluated using ImageJ (version 1.52i). Shed IECs were defined as high intensity DAPI signals (signals present after gating minimal threshold >200) while intact IECs were defined as lower intensity DAPI signals (signals present after gating minimal threshold >30). Shed and total IECs were enumerated through ImageJ ‘Analyzed Particles’ (>125inch^2^ pixel units; 0.10–1.00 circularity). Infected IECs and intracellular *Salmonella* were manually enumerated by eye. For all quantifications at least four images per condition were evaluated from two or more independent experiments.

### RNA extractions and quantitative real-time PCR

Immediately following euthanization of mice, cecal tissues were collected and placed in RNAlater (Qiagen), incubated at 4°C overnight, then stored at −80°C. Total RNA was extracted utilizing a RNeasy Mini Kit (Qiagen) according to the manufacturer's instructions. Total RNA was quantified utilizing a NanoDrop microvolume spectrophotometer, and corresponding cDNA was synthesized using 0.5μg of RNA with 5× All-In-One RT MasterMix (Abm). For the qPCR reaction, 5μl of a 1:10 dilution of cDNA was added to 10 μl Bio-Rad SsoFast EvaGreen Supermix with primers (final concentration, 300 nM; final volume, 20 μl), and qPCR was carried out using a Bio-Rad CFX Connect machine. Primers used were as follows: *Rplp0* (For– 5’ AGA TTC GGG ATA TGC TGT TGG C 3’; Rev– 5’ TCG GGT CCT AGA CCA GTG TTC 3’), *Casp11* (For– 5’ AAG CTG ATG CTG TCA AGC TG 3’; Rev– 5’ ATG ATT GTT GCA CCT TCA GGA 3’) and *Casp1* (For– 5’ CAA GGT GAT CAT TAT TCA GGC ATG 3’; Rev– 5’ CAA TGA AAA GTG AGC CCC TGA 3’). CFX Maestro software ver, 1.1 (Bio-Rad) was used for data quantification.

### Western blotting

Cell lysates were prepared as outlined previously [1]. Enteroids were resuspended in RIPA buffer with cOmplete protease inhibitors (Roche), sonicated, then centrifuged at 16,000xg for 20 min at 4°C. Total protein was estimated (660nm Protein Assay; Pierce) and 10 μg of whole cell lysate prepared according to manufacturer’s instructions in 1X Bolt LDS Sample Buffer with 1X Bolt Reducing Agent (Life Technologies) and heated at 70ºC for 10 min. Proteins were separated by Bolt 12% Bis-Tris Gel (Life Technologies), transferred to PVDF membrane (Life Technologies), followed by immunoblotting with mouse monoclonal anti-caspase-11 (p20 Flamy-1;1:1000; AdipoGen), mouse monoclonal anti-caspase-1 (p20 Casper-1;1:2000; AdipoGen), or mouse monoclonal anti-β-actin (G043; 1:2000; Applied Biological Materials), then with horse α-mouse IgG:HRP (7076; 1:2000; Cell Signaling Technologies).

### Enzyme-linked immunosorbent assay (ELISA)

Mice were infected as described above, 0.5–1 cm of the cecum excised, washed extensively in PBS then stored on ice in *ex vivo* secretion medium (FBS (2%, Sigma-Aldrich), RPMI (Gibco), Pen Strep (100 U/ml, Gibco), Sodium Pyruvate (1mM, Gibco), MEM non-essential amino acids (1X, Sigma-Aldrich), gentamicin (100μg/mL, Gibco). Streptomycin pretreated control ceca were collected from wildtype and *Casp1/11*^*−/−*^. Ceca and secretion medium were transferred under sterile conditions to a 24-well plate for 24h incubation. Media was then collected and centrifuged at 4C, supernatant collected and stored at -80C. Protein concentration was estimated as described above and 17 μg of total protein probed per well in duplicate according to the manufacturer’s instructions (murine IFN-γ ELISA MAX^™^ Deluxe Set; BioLegend).

### Statistical analysis

All results presented in this study are expressed as the mean values ± standard errors (SEM). Mann-Whitney U-test, student *t*-test and one-way ANOVA were performed using GraphPad Prism software, version 7.02 for Windows. A p-value of 0.05 or less was considered significant, with asterisks denoting significance in figures.

## Supporting information

S1 Fig Streptomycin-pretreated WT, *Casp11*^−/−^, *Casp1*^−/−^ and *Casp1*/*11*^−/−^ mice were orally infected with *S*. Typhimurium (3 × 10^6^ c.f.u.) and stool collected at 24 h and 48 h p.i. and plated to enumerate *Salmonella* shedding. Each symbol represents one animal. Mean and SEM are indicated. Results are from at least two independent experiments. Statistical significance was calculated using Mann-Whitney U-test (*p<0.05; **p<0.01; ***p<0.001).
(TIF)

S2 Fig Streptomycin-pretreated WT, *Casp11*^−/−^, *Casp1*^−/−^ and *Casp1*/*11*^−/−^ mice were orally infected with *S*. Typhimurium (3 × 10^6^ c.f.u.) and epithelial integrity in cecal tissues at 18 h p.i. scored blinded **(A)**. Representative H&E staining of cecal tissue from streptomycin-pretreated WT, *Casp11*^−/−^, *Casp1*^−/−^ and *Casp1*/*11*^−/−^ mice at 18 h p.i. **(B)**. Arrows denote IECs that are actively shedding or have been shed. Original magnification ×200; scale bars 100 μm. Statistical significance was calculated using Mann-Whitney U-test (*p<0.05; ***p<0.001).
(TIF)

S3 Fig Cecal enteroid monolayers derived from WT, *Casp11*^−/−^, *Casp1*^−/−^ and *Casp1*/*11*^−/−^ mice were infected with either wildtype *S*. Typhimurium (SL1344) or mCherry-*S*. Typhimurium (mCherry) (MOI of 50) for 10 h, then monolayers lysed and bacterial counts enumerated. Results are from three independent experiments. Statistical significance was calculated using student *t*-test with no significant difference between SL1344 and mCherry c.f.u. for each monolayer genotype.
(TIF)

S4 Fig Cecal enteroid monolayers derived from WT, *Casp11*^−/−^, *Casp1*^−/−^ and *Casp1*/*11*^−/−^ mice were infected with mCherry-*S*. Typhimurium (MOI of 50) and the percentage of shed infected IECs at 10 h p.i. enumerated. Results are from at least 400 IECs from two independent experiments. Statistical significance was calculated using Mann-Whitney U-test n.s. p>0.05; **p<0.01; ***p<0.001.
(TIF)

S5 Fig Cecal enteroid monolayers were infected with mCherry-*S*. Typhimurium (MOI of 50) and inflammatory caspase activity assessed at 10 h p.i. Over-exposed fluorescence image of *Casp1*/*11*^−/−^ monolayer depicting an overall lack of inflammatory caspase activity (660-YVAD-FMK activity; green; 10X exposure time compared to Fig 3) in shedding IECs heavily infected with *Salmonella* (red), actin (white) and DNA (blue). Original magnification ×400; scale bars 25μm.
(TIF)

S6 Fig Streptomycin-pretreated WT, *Casp11*^−/−^, *Casp1*^−/−^ and *Casp1*/*11*^−/−^ mice were orally infected with *S*. Typhimurium (3 × 10^6^ c.f.u.) for 18 h and 72 h p.i., ceca collected and transferred to secretion media for 24 h. Streptomycin-pretreated WT and *Casp1/11*^−/−^ uninfected ceca were also collected as controls (Ctrl). *Ex vivo* secretions were measured by ELISA for murine IFN-γ. Each symbol represents one animal. Mean and SEM are indicated. Results are from at least two independent experiments. Statistical significance was calculated using student *t*-test *p<0.05; ***p<0.001.
(TIF)

S7 Fig Cecal enteroid monolayers from WT, *Casp11*^−/−^, *Casp1*^−/−^ and *Casp1*/*11*^−/−^ mice were either pretreated with IFN-γ (10 ng/mL) or vehicle control 16 h prior to infection with mCherry-*S*. Typhimurium (MOI of 50) and the percentage of shed/shedding IECs (from five blinded fields of view from two independent experiments) at 10 h p.i. enumerated. Statistical significance was calculated using one-way ANOVA; no significant difference was determined between samples.
(TIF)

S1 Data Excel spreadsheet containing, in separate sheets, the underlying numerical data and statistical analysis for Figure panels 1A, 1B, 1D, 1E, 1F, 2B, 2C, 2E, 2F, 4A, 4B, 4C, S1, S2, S3, S4, S6 and S7. (XLSX)
