# Supplementary figures and images for: Intestinal restriction of Salmonella Typhimurium requires caspase-1 and caspase-11 epithelial intrinsic inflammasomes
Source: PLoS Pathog. 2020 Apr 13;16(4):e1008498. doi: 10.1371/journal.ppat.1008498 (PMC7179941; doi:10.1371/journal.ppat.1008498)

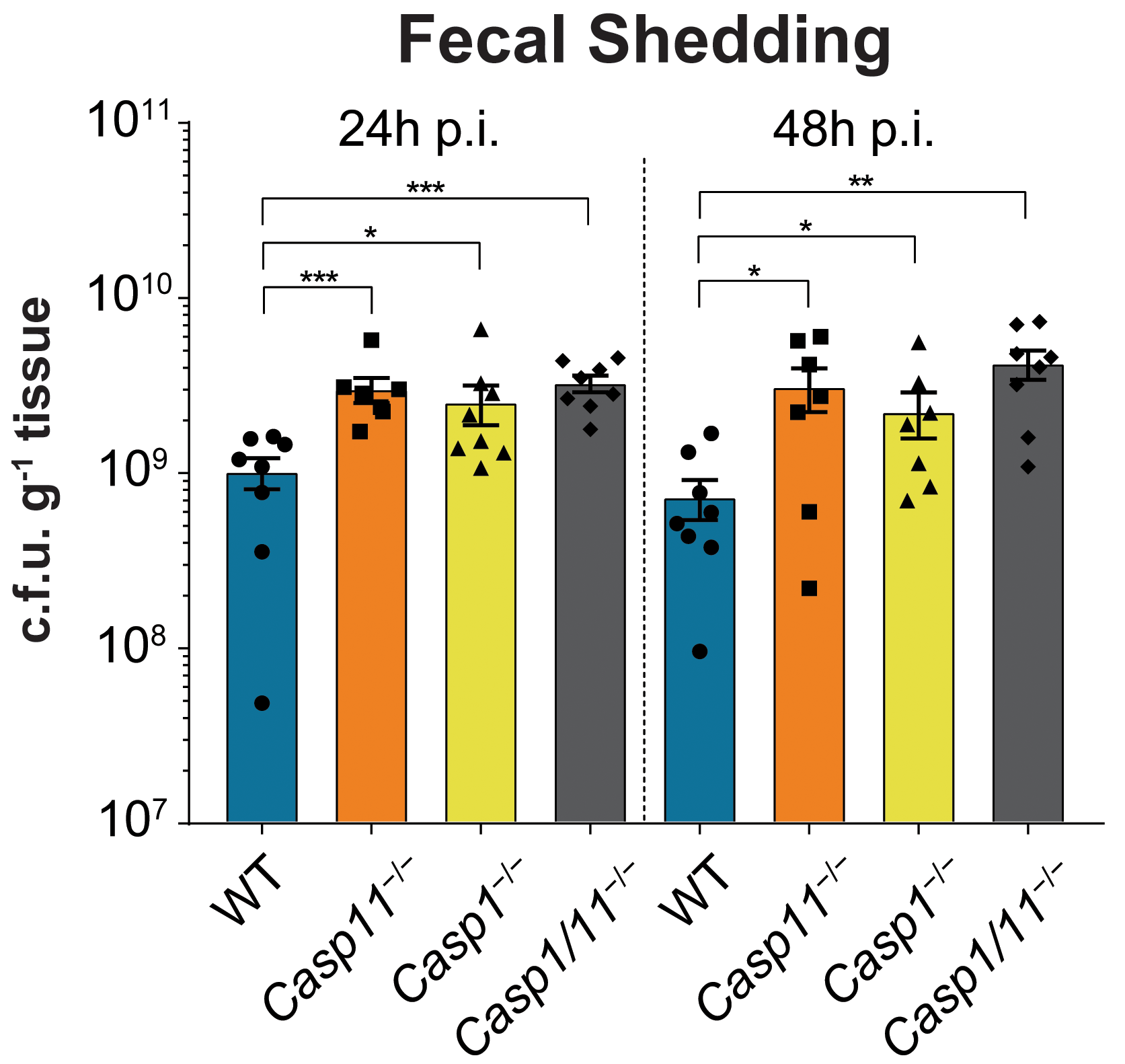

Supplement: S1 Fig — Each symbol represents one animal. Mean and SEM are indicated. Results are from at least two independent experiments. Statistical significance was calculated using Mann-Whitney U-test (*p<0.05; **p<0.01; ***p<0.001). (TIF) [file ppat.1008498.s001.tif]

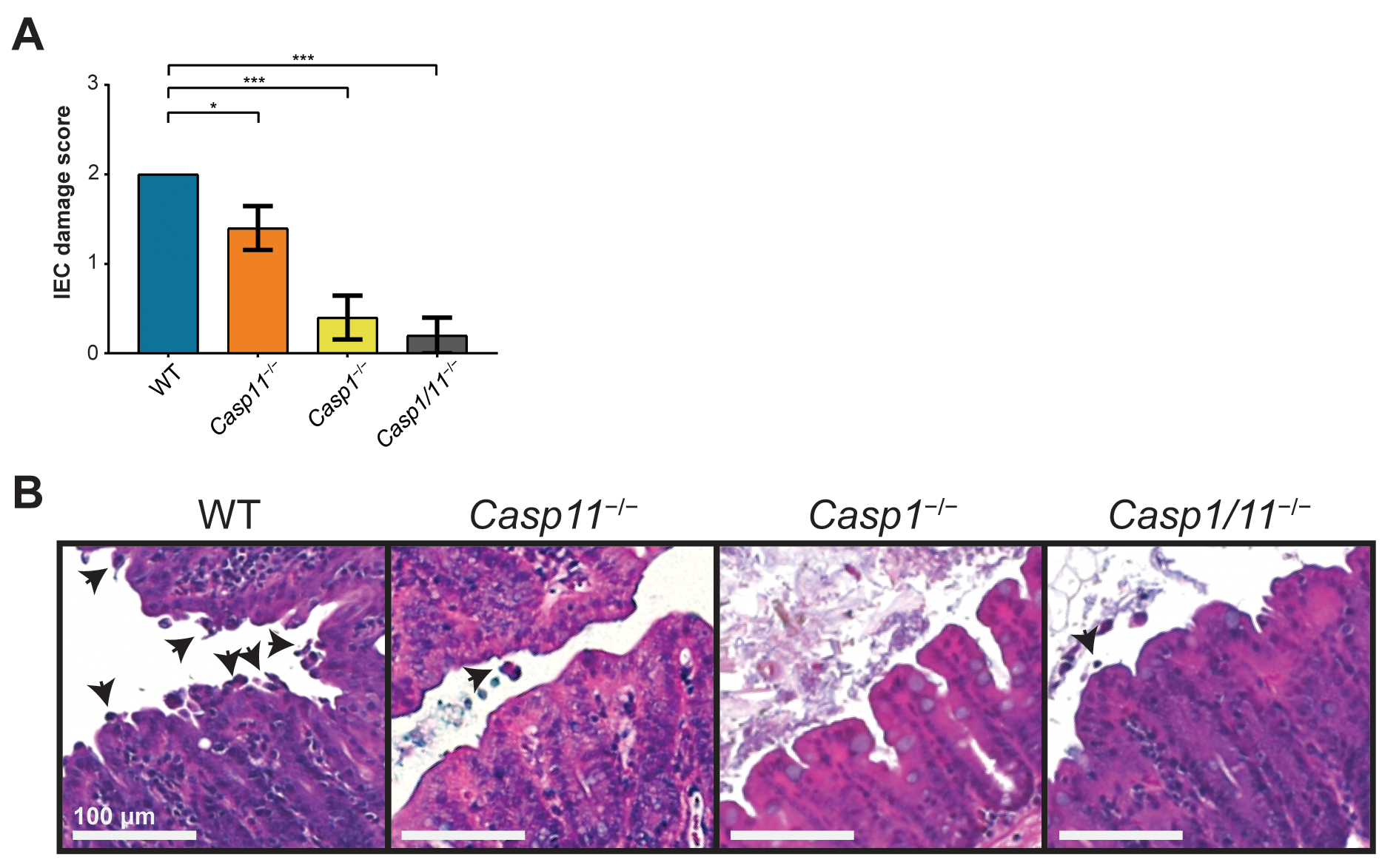

Supplement: S2 Fig — Streptomycin-pretreated WT, Casp11−/−, Casp1−/− and Casp1/11−/− mice were orally infected with S. Typhimurium (3 × 10^6^ c.f.u.) and epithelial integrity in cecal tissues at 18 h p.i. scored blinded (A). Representative H&E staining of cecal tissue from streptomycin-pretreated WT, Casp11−/−, Casp1−/− and Casp1/11−/− mice at 18 h p.i. (B). Arrows denote IECs that are actively shedding or have been shed. Original magnification ×200; scale bars 100 μm. Statistical significance was calculated using Mann-Whitney U-test (*p<0.05; ***p<0.001). (TIF) [file ppat.1008498.s002.tif]

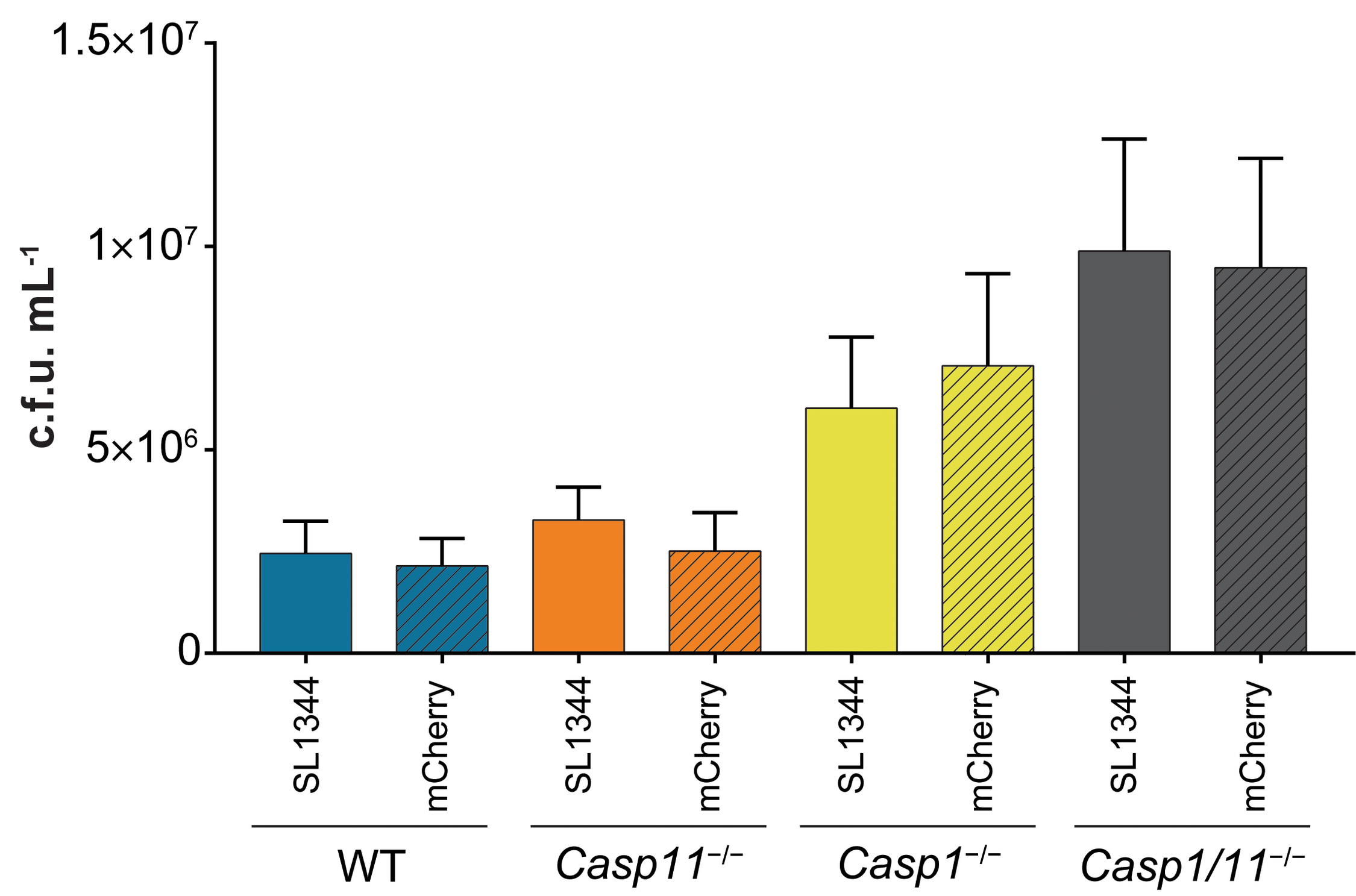

Supplement: S3 Fig — Statistical significance was calculated using student t-test with no significant difference between SL1344 and mCherry c.f.u. for each monolayer genotype. (TIF) [file ppat.1008498.s003.tif]

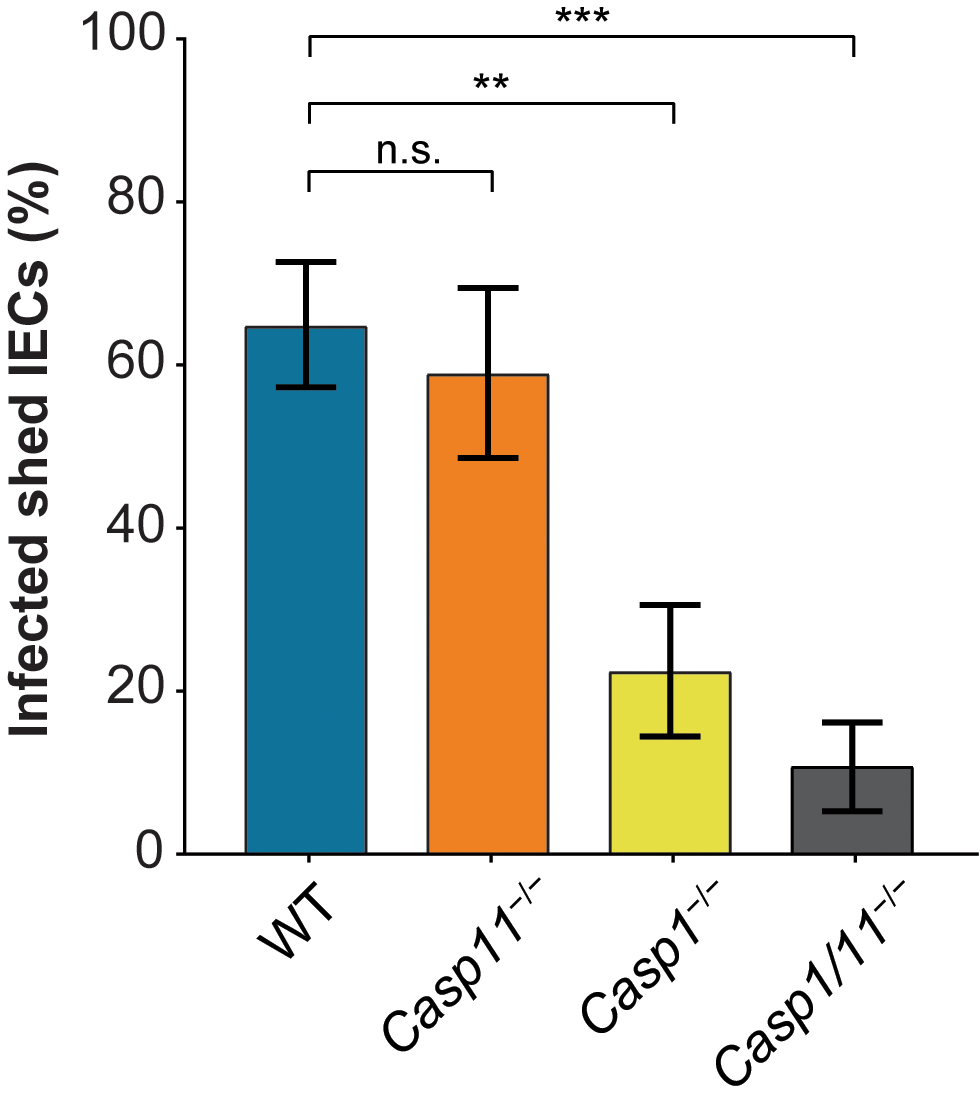

Supplement: S4 Fig — Results are from at least 400 IECs from two independent experiments. Statistical significance was calculated using Mann-Whitney U-test n.s. p>0.05; **p<0.01; ***p<0.001. (TIF) [file ppat.1008498.s004.tif]

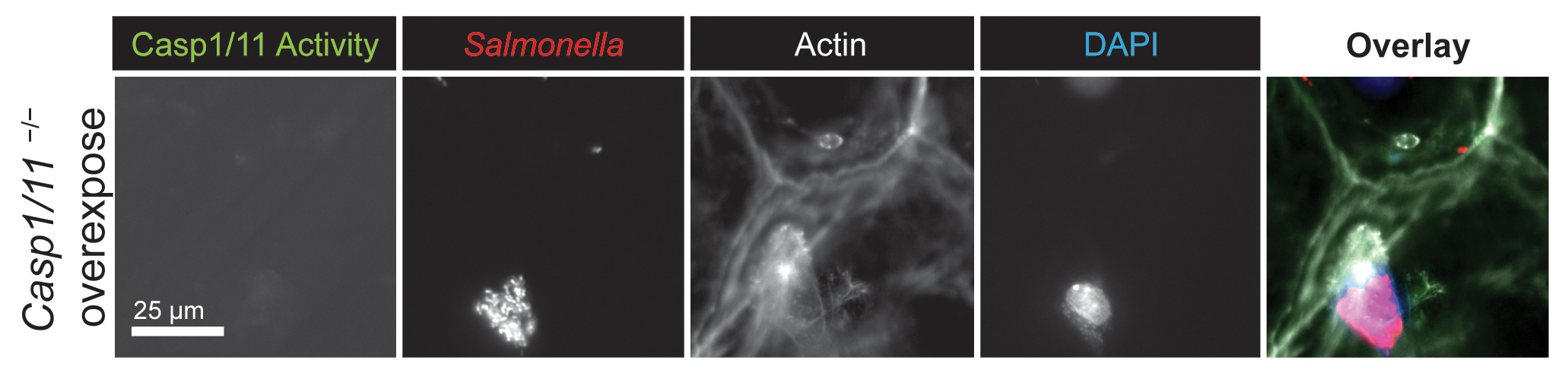

Supplement: S5 Fig — Over-exposed fluorescence image of Casp1/11−/− monolayer depicting an overall lack of inflammatory caspase activity (660-YVAD-FMK activity; green; 10X exposure time compared to Fig 3) in shedding IECs heavily infected with Salmonella (red), actin (white) and DNA (blue). Original magnification ×400; scale bars 25μm. (TIF) [file ppat.1008498.s005.tif]

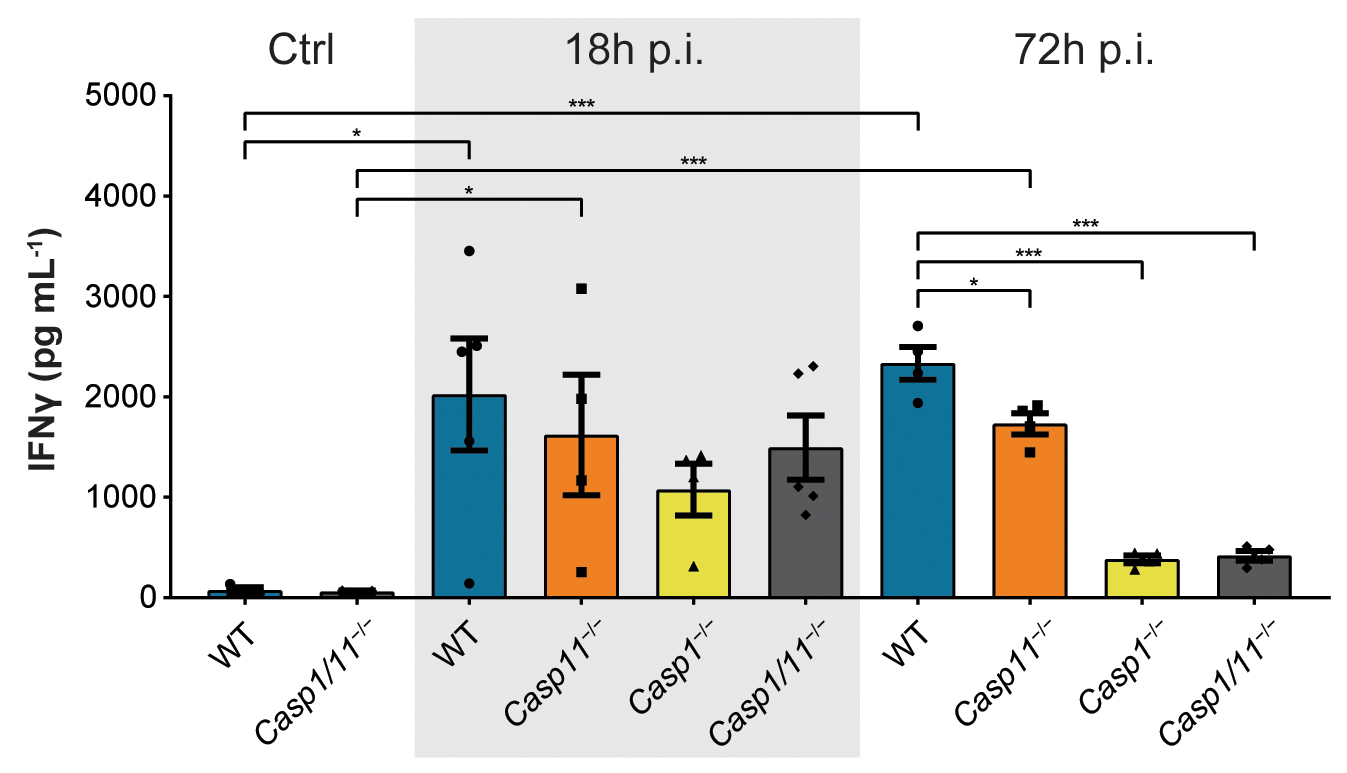

Supplement: S6 Fig — Streptomycin-pretreated WT and Casp1/11−/− uninfected ceca were also collected as controls (Ctrl). Ex vivo secretions were measured by ELISA for murine IFN-γ. Each symbol represents one animal. Mean and SEM are indicated. Results are from at least two independent experiments. Statistical significance was calculated using student t-test *p<0.05; ***p<0.001. (TIF) [file ppat.1008498.s006.tif]

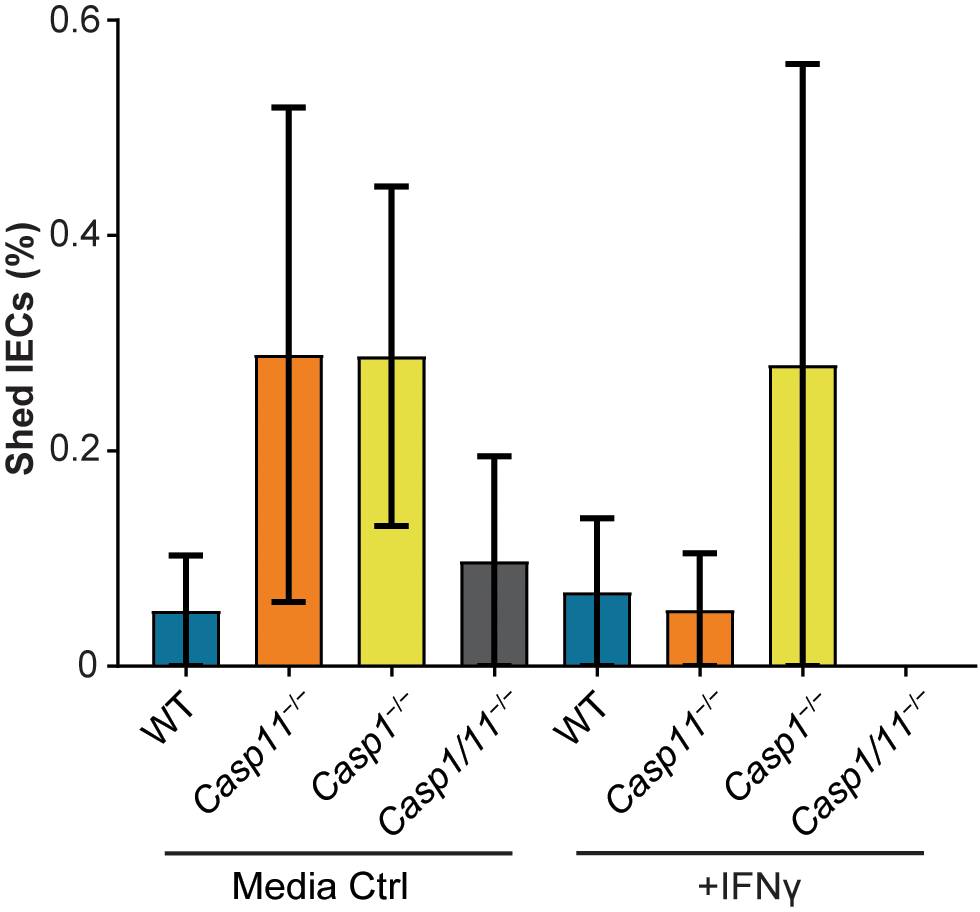

Supplement: S7 Fig — Statistical significance was calculated using one-way ANOVA; no significant difference was determined between samples. (TIF) [file ppat.1008498.s007.tif]
